# Supplementary material for: Association between oral microbiome and seven types of cancers in East Asian population: a two-sample Mendelian randomization analysis
Source: Front Mol Biosci. 2023 Nov 21;10:1327893. doi: 10.3389/fmolb.2023.1327893 (PMC10702768; doi:10.3389/fmolb.2023.1327893)
Supplement: Supplementary file 1 [file DataSheet1.ZIP › Supplementary Materials/MR plots of saliva/saliva═╝/Breast cancer/pheno.3029_to_breast cancer_forest.pdf]

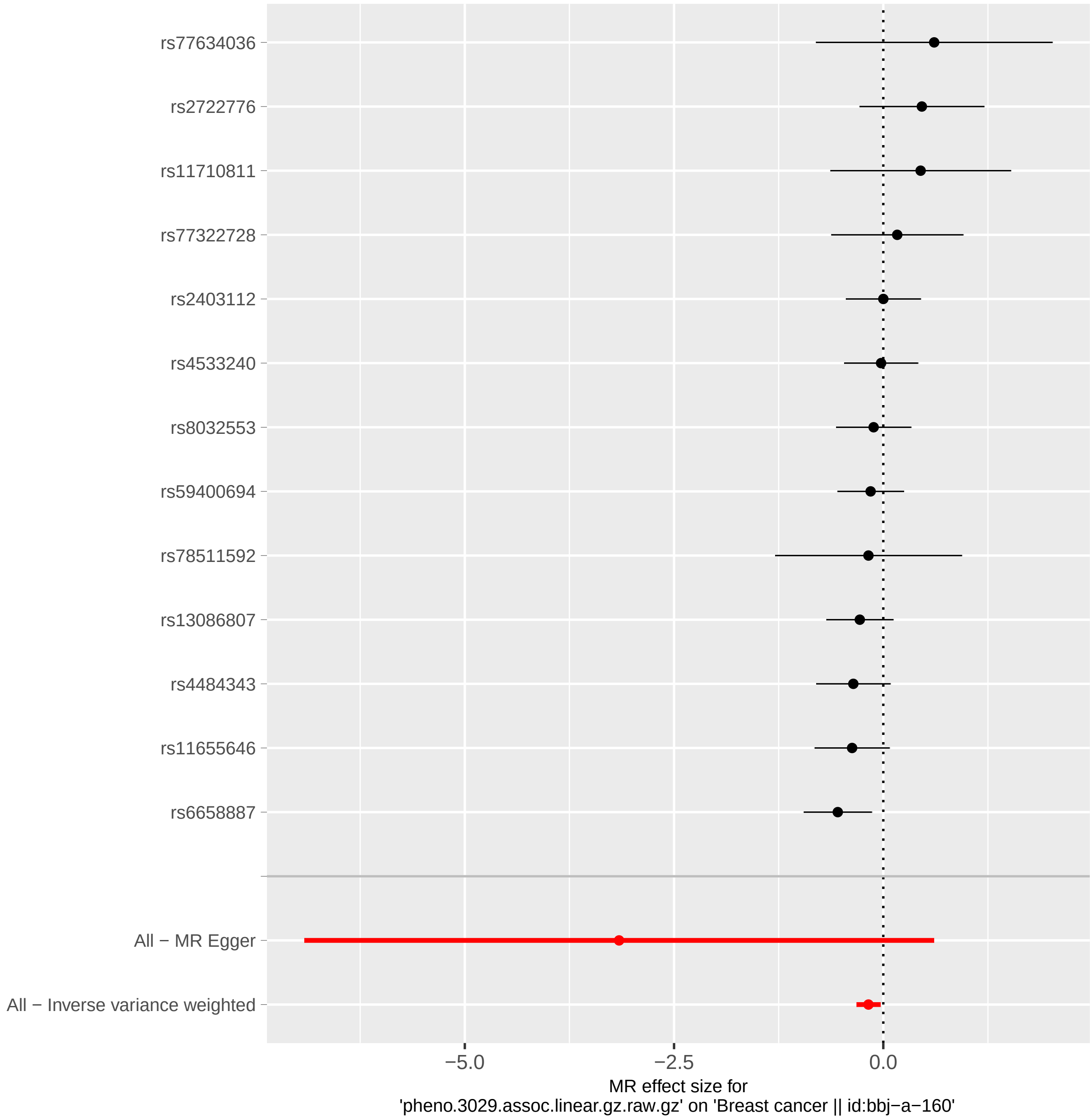
rs77634036
rs2722776
rs11710811
rs77322728
rs2403112
rs4533240
rs8032553
rs59400694
rs78511592
rs13086807
rs4484343
rs11655646
rs6658887
All − MR Egger
All − Inverse variance weighted
−5.0
−2.5
0.0
MR effect size for
'pheno.3029.assoc.linear.gz.raw.gz' on 'Breast cancer || id:bbj−a−160'
